# The Relationship between Insomnia and the Pathophysiology of Major Depressive Disorder: An Evaluation of a Broad Selection of Serum and Urine Biomarkers

**DOI:** 10.3390/ijms24098437

**Published:** 2023-05-08

**Authors:** Tina Drinčić, Jens H. van Dalfsen, Jeanine Kamphuis, Mike C. Jentsch, Sjoerd M. van Belkum, Marcus J. M. Meddens, Brenda W. J. H. Penninx, Robert A. Schoevers

**Affiliations:** 1 Department of Psychiatry, University Medical Centre Groningen, Hanzeplein 1, 9713 RB Groningen, The Netherlands; 2 Independent Researcher, 7251 RT Vorden, The Netherlands; 3 Department of Psychiatry, Amsterdam University Medical Centre (VUmc), De Boelelaan 1118, 1081 HZ Amsterdam, The Netherlands

**Keywords:** major depressive disorder, insomnia, pathophysiology, biomarkers

## Abstract

Insomnia exhibits a clinically relevant relationship with major depressive disorder (MDD). Increasing evidence suggests that insomnia is associated with neurobiological alterations that resemble the pathophysiology of MDD. However, research in a clinical population is limited. The present study, therefore, aimed to investigate the relationship between insomnia and the main pathophysiological mechanisms of MDD in a clinical sample of individuals with MDD. Data were extracted from three cohorts (*N* = 227) and included an evaluation of depression severity (Quick Inventory of Depressive Symptomatology, QIDS-SR_16_) and insomnia severity (QIDS-SR_16_ insomnia items) as well as serum and urine assessments of 24 immunologic (e.g., tumour necrosis factor α receptor 2 and calprotectin), neurotrophic (e.g., brain-derived neurotrophic factor and epidermal growth factor), neuroendocrine (e.g., cortisol and aldosterone), neuropeptide (i.e., substance P), and metabolic (e.g., leptin and acetyl-L-carnitine) biomarkers. Linear regression analyses evaluating the association between insomnia severity and biomarker levels were conducted with and without controlling for depression severity (*M* = 17.32), antidepressant use (18.9%), gender (59.0% female; 40.5% male), age (*M* = 42.04), and the cohort of origin. The results demonstrated no significant associations between insomnia severity and biomarker levels. In conclusion, for the included biomarkers, current findings reveal no contribution of insomnia to the clinical pathophysiology of MDD.

## 1. Introduction

Insomnia and major depressive disorder (MDD) are closely related. Insomnia is one of the primary symptoms of MDD with around 40–75% of the MDD population meeting the diagnostic criteria for clinical insomnia [1,2,3]. In addition to being a core symptom, insomnia has been widely recognised as an independent contributor to the aetiology of MDD. Recent meta-analyses have revealed that individuals with insomnia are two to three times as likely to develop MDD relative to people without sleep disturbances [4,5]. Clinical evidence further demonstrates that insomnia negatively impacts the clinical course of MDD, including a prolonged duration and greater severity of the depressive episode and an increased risk of relapse following successful treatment [6,7]. In line with these findings, systematic reviews conclude that the treatment of comorbid insomnia may improve treatment outcomes in MDD [8,9]. While the clinical relevance of insomnia has been well established, it remains unclear how insomnia relates to the underlying neurobiology of MDD.

The pathophysiology of MDD involves a diverse range of neurobiological mechanisms. This includes alterations in immunologic [10,11], neurotrophic [12,13], neuroendocrine [14], neuropeptide [15], and metabolic [16] functioning. Although these pathophysiological hypotheses have found support in studies comparing individuals with MDD to healthy controls, this field of research has yielded yet inconclusive results [17,18,19]. While these inconsistent findings may relate to methodological aspects in biomarker assessment [20], they also presumably reflect the marked heterogeneity in MDD. The diagnostic classification of MDD, according to the Diagnostic and Statistical Manual of Mental Disorders fifth edition (DSM−5) [21], is based on a diverse range of symptoms. According to this classification, different combinations of sometimes even opposing symptoms may result in an MDD diagnosis [22]. Hence, MDD is a heterogenous disorder in both clinical presentation [22] and underlying pathophysiology [23]. Evaluating the association between symptoms with known clinical relevance, such as insomnia, and presumed pathophysiological mechanisms could therefore be crucial to advance the neurobiological understanding of MDD [24].

Previous research provides valuable support for an association between insomnia and neurobiological alterations that resemble the pathophysiology of MDD. This includes increased inflammation [25], decreased brain-derived neurotrophic factor (BDNF) [26], increased hypothalamic-pituitary-adrenal (HPA) axis (re)activity [27,28] as well as changes in neuropeptides [29] and metabolic functioning [30]. While these findings elucidate an association between insomnia and the pathophysiological mechanisms of MDD, research in individuals with MDD is limited. The initial findings on the level of inflammation have supported an association between insomnia and the underlying neurobiological processes of MDD in a clinical population [31,32,33,34,35]. It remains unclear, however, how insomnia relates to the broad spectrum of molecular mechanisms implicated in the clinical pathophysiology of MDD.

The present study, therefore, aimed to explore the relationship between insomnia and a diverse selection of immunologic (e.g., tumour necrosis factor-α receptor 2 and calprotectin), neurotrophic (e.g., brain-derived neurotrophic factor and epidermal growth factor), neuroendocrine (e.g., cortisol and aldosterone), neuropeptide (i.e., substance P), and metabolic (e.g., leptin and acetyl-L-carnitine) biomarkers in a clinical sample of individuals with MDD. Utilizing data from three cohorts in which blood and urine samples have been collected and analysed using the same assays, it was investigated whether the severity of insomnia is associated with biomarker levels. It was further examined whether depression severity, antidepressant use, gender, age, and the cohort of origin confounded this relationship. It is hypothesised that increasing insomnia severity exacerbates the neurobiological abnormalities associated with MDD, reflecting the potential involvement of insomnia in the clinical pathophysiology of MDD.

## 2. Results

### 2.1. Data

From the different research cohorts (see: Section 4.1), the total sample consisted of 227 individuals with MDD with available data on both insomnia severity and at least 1 biomarker level, making them eligible for inclusion in the univariate analyses. In this sample, the data on all relevant covariates were available for 189 individuals who were therefore included in the multivariate analyses. Notably, the number of participants included in the analyses varied depending on the availability of individual biomarker levels. The number of outliers for a given biomarker level ranged from 0 to 5, with no outliers detected for 13 biomarker levels. A total of 34 data points were identified as outliers and therefore excluded from all analyses. The actual number of participants included in each regression model is reported in the tables.

### 2.2. Demographic and Clinical Characteristics

The demographic and clinical characteristics are presented in Table 1. The total sample of individuals with MDD (*N* = 227) included 134 women (59.0%) and 92 men (40.5%). The age of participants ranged from 19 to 76, with a mean of 42.04 years (*SD* = 12.63). Current antidepressant use was present in 43 participants (18.9%). The total self-rated Quick Inventory of Depressive Symptomatology (QIDS-SR_16_) score ranged from 4 to 27. The mean QIDS-SR_16_ score was 17.32 (*SD* = 4.91), indicating that, on average, participants were experiencing severe depression. The mean QIDS-SR_16_ score with the sleep items excluded was 14.86 (*SD* = 4.57). The mean insomnia severity sub-score was 4.69 (*SD* = 2.47), ranging from 0 to 9. The duration of the current MDD episode was available for 177 participants, with 42.3% of the total sample experiencing an episode for less than a month, 8.8% for between one and six months, 4.8% for between six months and a year, and 21.6% for longer than a year. The total number of episodes ranged from 1 to 96, with a median of 2 and an interquartile range (IQR) from 1 to 5.

### 2.3. The Influence of Insomnia on Biomarker Levels

#### 2.3.1. Univariate Analyses

The results of the univariate linear regression analyses are reported in Table 2 (serum biomarker levels) and Table 3 (urine biomarker levels). No significant associations between insomnia severity and the biomarker levels were found when controlling for multiple testing. Notably, prior to adjusting for multiple testing, a significant negative association between insomnia severity and serum resistin levels (*b* = −0.030; 95% confidence interval (CI) [−0.051, −0.009]; β = −0.186; unadjusted *p* = 0.006; adjusted *p* = 0.151; *R*^2^ = 0.035) and a significant positive association been insomnia severity and serum acetyl-L-carnitine levels (*b* = 0.039; 95% CI [.010, 0.068]; β = 0.178; unadjusted *p* = 0.009; adjusted *p* = 0.151; *R*^2^ = 0.032) were observed.

#### 2.3.2. Multivariate Analyses

The results of the multivariate linear regression analyses are reported in Table 4 (serum biomarker levels) and Table 5 (urine biomarker levels). The regression statistics of each covariate are further provided in Supplementary Table S1 (serum biomarker levels) and Supplementary Table S2 (urine biomarker levels). Controlling for depression severity, antidepressant use, gender, age, and the cohort of origin, no significant associations between insomnia severity and the biomarker levels were observed when adjusting for multiple testing. Notably, prior to adjusting for multiple testing, a significant negative association between insomnia severity and serum endothelin-1 levels (*b* = −0.019; 95% CI [−0.037, −0.002]; β = −0.183; unadjusted *p* = 0.033; adjusted *p* = 0.528; *R*^2^ = 0.119) was found, and the significant positive association with serum acetyl-L-carnitine levels observed in the univariate analyses remained significant after controlling for relevant covariates (*b* = 0.036; 95% CI [.000, 0.073]; β = 0.167; unadjusted *p* = 0.049; adjusted *p* = 0.528; *R*^2^ = 0.134).

## 3. Discussion

The aim of the present study was to investigate the relationship between insomnia severity and the immunologic, neurotrophic, neuroendocrine, neuropeptide, and metabolic processes underlying MDD in a clinical sample of individuals with MDD. In contrast to the hypothesis, the results demonstrated no significant associations between insomnia and the biomarker levels for insomnia severity as a single predictor (univariate models) nor for insomnia severity corrected for depression severity, antidepressant use, gender, age, and the cohort of origin (multivariate models). Taken together, for the included biomarkers current findings do not support a profound influence of insomnia on the clinical pathophysiology of MDD.

The relationship between sleep disturbances and the proinflammatory markers represents the most widely explored relationship in a clinical sample of individuals with MDD relative to other pathophysiological mechanisms. The absence of a significant association between insomnia and immunologic biomarkers observed in the present study adds to the inconsistent findings of previous research. Multiple studies in individuals with MDD have demonstrated that sleep disturbances were associated with higher levels of circulating C-reactive protein (CRP) [31,32,33], serum tumour necrosis factor (TNF) α [34], interleukin(IL)−6, interferon(IFN)-α2, and IFN-γ [35]. However, this has not been consistently observed in a clinical sample for CRP, TNFα, and IL−6 [36]. Research on CRP suggests that several factors might confound this relationship as the effect of sleep disturbances was not maintained after controlling for relevant covariates in some [32], but not all [31], studies in individuals with MDD. Importantly, the research in non-depressed individuals found more conclusive relationships between insomnia and proinflammatory markers. A recent meta-analysis demonstrated that sleep disturbances are associated with higher levels of CRP and IL−6 [37], which also represent the most consistently identified immunologic abnormalities in MDD [18,20,38]. However, there is little evidence that inflammatory cytokines serve as valuable diagnostic biomarkers of insomnia [39]. Taken together, while studies in healthy populations suggest that sleep disturbances may exacerbate the immunologic abnormalities associated with MDD, the findings in clinical samples are inconsistent. Nonetheless, since previous studies reporting a significant association between insomnia and inflammation included biomarkers that were not included in the present study, it cannot be excluded that an association between insomnia and these biomarkers may exist in a clinical population.

Similar to the findings on immunologic biomarkers, the present study did not reveal an effect of insomnia on the levels of neurotrophic factors and neuropeptides. Previous research concerning the relationship between sleep disturbances and neurotrophic factors in clinical samples is limited and inconclusive. One study found that poorer sleep quality was associated with increased serum BDNF levels in MDD [40], whereas another relatively small intervention study observed that improvements in sleep quality were positively correlated with BDNF concentrations [41]. In contrast to the inconsistent findings in clinical populations, a meta-analysis of studies in healthy participants demonstrated that sleep disturbances were associated with lower levels of BDNF [26]. In line with this observation, there is evidence that BDNF may be a useful predictor of insomnia [39]. Although the direction of this association resembles the BDNF alterations observed in MDD [42], the present study could not verify a potential contribution of insomnia to neurotrophic abnormalities in a clinical sample. Concerning neuropeptides, the lack of a significant association between insomnia and substance P accords with a previous study on 186 individuals with MDD, which revealed that sleep disturbances were not correlated with the plasma levels of substance P and four other neuropeptides [43].

The relationship between insomnia and neuroendocrine biomarkers in an MDD population has not been studied extensively in previous research. The absence of a relationship observed in the present study is, however, in contrast to a recent finding of decreased levels of salivary cortisol in individuals with MDD and poor sleep quality [40]. The effect of insomnia on neuroendocrine function has been more elaborately investigated in non-depressed populations. Although serum cortisol levels seem to have a low diagnostic accuracy for insomnia [39], various meta-analyses and systematic reviews have identified that disturbed sleep is associated with elevated basal cortisol levels (e.g., [44]) as well as an increased stress reactivity of the HPA-axis (e.g., [27]). While these findings bare resemblance to neuroendocrine changes found in individuals with MDD relative to healthy controls [45,46], it is important to note that long-lasting MDD has also been associated with lower cortisol levels [47,48,49]. This may be due to adrenal exhaustion or the decreased feedback sensitivity of the HPA-axis due to chronic stress [50]. For this reason, the role of insomnia in increasing HPA-axis activity may not be observable in the more advanced stages of MDD. This could explain the absence of a significant association, as observed in the present study.

The present study did not observe an effect of insomnia severity on the levels of metabolic biomarkers. This relationship has not been previously examined in a clinical population of individuals with MDD, and research in healthy participants yielded inconclusive findings. While it has been shown that sleep restriction is related to a reduction in leptin levels [30], an association between insomnia and increased leptin has also been reported [51]. Since both leptin [16] and acetyl-L-carnitine [52] appear to be decreased in MDD, a negative association with insomnia would support a contribution of insomnia to metabolic changes in MDD. However, the present study does not confirm such a relationship in MDD.

To summarize, the current findings do not support a profound influence of insomnia on the pathophysiology of MDD in a clinical MDD population. This contrasts with previous findings in non-clinical populations, revealing that sleep disturbances are associated with a range of immunologic [18,20,37,38,42], neurotrophic [26,42], and neuroendocrine [27,44,45,46] alterations that resemble the pathophysiology of MDD. While more research is needed, these conflicting findings might suggest that the relationship between insomnia and pathophysiological mechanisms may be especially relevant for the aetiology of MDD. This postulation is supported by studies evaluating the mediating role of these pathophysiological processes in the relationship between sleep disturbance and the development of depressive symptoms [53,54]. However, based on the current findings it is unlikely that the influence of insomnia on the clinical course of MDD [6,7] relates to alterations in immunologic, neurotrophic, and neuroendocrine functioning.

### Limitations and Future Directions

The main strength of the present study is the inclusion of a broad panel of high-quality biomarker assessments that cover all the neurobiological hypotheses of MDD. The present study is, however, subjected to the following limitations. First, the relatively small sample size may have limited the ability to observe significant differences. Hence, the high number of included biomarkers requires a relatively large sample to have sufficient statistical power to detect significant associations after adjusting for multiple testing. Nonetheless, unadjusted analyses also did not reveal significant associations in the expected direction, indicating that insomnia is unlikely to exacerbate the pathophysiology of MDD in a clinical sample. Second, the laboratory assessments did not include all biomarkers implicated in the pathophysiology of MDD. For future studies, it is therefore recommended to evaluate a narrow selection of biomarkers including CRP [18,37,38], IL−6 [18,20,37,38], BDNF [26,42], and more detailed measures of HPA-axis (re)activity [27,44,45,46]. Third, while the insomnia items of the QIDS-SR_16_ [36,55,56,57] and other depression scales [58,59,60] are commonly used to assess insomnia severity, this approach allows for relatively little variation compared to validated insomnia questionnaires. Future studies should therefore incorporate questionnaires that are specifically developed for the assessment of insomnia severity. Fourth, the time of biometric sample collection was only standardised in a part of the total sample. Hence, the accuracy of the reported associations may have been affected by diurnal fluctuations of biomarker levels [61]. Fifth, while the effect of insomnia was adjusted for important covariates in the multivariate analyses, information on other potentially relevant confounders known to affect biomarker levels, such as BMI [36,62] and the presence of obstructive sleep apnoea [63,64,65], was not available, and it is advised to control for these factors in future studies. Given the profound differences in studies evaluating the association between insomnia and pathophysiological mechanisms in healthy and clinical populations, further research should evaluate the presumed relevance of this relationship for the involvement of insomnia in the aetiology of MDD.

## 4. Materials and Methods

### 4.1. Data Source

The data used in the present study comprise previously collected data retrieved from three different studies: Pieken in de Delta Oost Nederland (PIDON) [66], transcranial pulsed electromagnetic fields (tPEMF) [67], and the Mood Treatment with Antidepressants or Running (MOTAR) study [68,69]. The baseline measurement included in the original investigations was utilized for the performed analyses (see Section 4.4). This includes an assessment of clinical depression (see Section 4.2) and depression symptoms (see Section 4.3.1). Biomarker levels were determined in the blood and urine samples collected at baseline as part of the initial study procedures of the included studies. Notably, biomarker assessment has been previously performed using the same assay in all research cohorts (see Section 4.3.3).

Data of the PIDON cohort (*n* = 38) were collected in a study aimed at the development of an algorithm to calculate a depression probability score differentiating individuals with MDD from healthy controls based on biomarker measurements [66]. Participants were recruited in the Netherlands via general practitioners, psychiatric care organisations, and newspaper advertisements. The study aimed to provide evidence for an association between a cluster of biomarkers and an MDD diagnosis. Blood and urine sampling was performed on the same day as the clinical interviews. Blood samples were collected between 8:00 AM and 6:00 PM. Urine samples were collected in the morning from the first urination. The tPEMF cohort (*n* = 56) comprised a sample of individuals with MDD included in a double-blind, randomised control trial comparing transcranial pulsed electromagnetic fields (tPEMF) to sham treatment [67]. Patients were recruited from major mental health care institutions in the northern part of the Netherlands and through media coverage. The primary aim was to investigate the antidepressant effect of tPEMF in treatment-resistant depression. Clinical interviews and biological sampling were performed on the same day in the morning or early afternoon. The MOTAR study comprises a 16-week intervention study comparing antidepressant medication and running therapy [68,69]. Individuals with MDD (*n* = 133) were recruited from a mental health organisation in the surroundings of Amsterdam. The main objective of the study was to investigate the effect of antidepressant medication and running therapy on depressive symptoms and biological ageing. Fasting blood and urine samples were collected between 8:30 and 9:30 AM during baseline assessment, which included a clinical interview and the completion of questionnaires.

### 4.2. Study Population

The study population (*N* = 227) comprises individuals with current MDD included in the research cohorts aged between 18 and 80 years. MDD diagnoses according to DSM-IV criteria were obtained using either the Mini International Neuropsychiatric Interview (MINI) [70] in the tPEMF [67] and PIDON [66] cohorts or the Composite International Diagnostic Interview (CIDI) [71] in the MOTAR cohort [68,69].

Shared inclusion criteria of the different cohorts include a current MDD diagnosis ascertained by the MINI or the CIDI and age 18–80. Importantly, the tPEMF cohort only included individuals with at least moderately severe depression who were non-responsive to one or more antidepressants provided for at least four weeks in an adequate dose. Shared exclusion criteria comprised pregnancy and the presence of another primary psychiatric disorder. Additionally, the tPEMF and MOTAR cohorts excluded individuals with evidence of (high) suicidal risk as well as individuals who recently or currently used other psychotropic medication. In the tPEMF cohort, individuals with a change in antidepressant medication in the four weeks prior to the study were additionally excluded. In the MOTAR cohort, individuals who used antidepressants in the previous two weeks, individuals who exercised regularly more than once a week, and individuals with medical contraindications to running therapy or antidepressants were excluded.

In the present study, participants were excluded from the univariate analysis if data were missing regarding any item on the scales used to measure insomnia symptoms (see Section 4.3.2) or if no data on biomarker levels were available. Patients were additionally excluded from the multivariate analysis if data were missing regarding any of the corresponding covariates.

### 4.3. Measurements

#### 4.3.1. Depression

To assess the severity of depression, the self-rated Inventory of Depressive Symptomatology (IDS-SR_30_) [72] was used in the tPEMF [67] and MOTAR [68] cohorts, and the self-rated Quick Inventory of Depressive Symptomatology (QIDS-SR_16_) [73] was used in the PIDON cohort [67]. The QIDS-SR_16_ consists of items analogous to IDS-SR_30_. The total IDS-SR_30_ score was therefore converted to the total QIDS-SR_16_ score according to previously developed metrics [73] in the tPEMF and MOTAR cohorts.

The IDS-SR_30_ is a well-validated questionnaire that measures depression severity [72]. It inquires about all DSM-IV [74] melancholic and atypical criterion symptoms during the previous week and shows good internal consistency and interrater reliability [72]. As changes in appetite and weight are scored separately depending on the direction of change, the total score of the IDS-SR_30_ is based on 28 of 30 items, resulting in total scores ranging from 0 to 84. Each item measures one symptom construct and is scored as a Likert scale with points ranging from 0 to 3, with higher scores indicating greater depression severity. The QIDS-SR_16_ is based on a subset of the IDS-SR_30_, including 16 analogous items relevant to 9 DSM-IV [74] symptom domains [73]. It is well validated, shows high internal consistency, and its total score has been shown to highly correlate with the IDS-SR_30_ total score [73]. The total score is calculated by adding points of the maximally scored item in each symptom domain, yielding total scores ranging from 0 to 27. Higher scores are indicative of greater depression severity.

Items 1–4 of both the IDS-SR_30_ and the QIDS-SR_16_ relate to the sleep disturbance symptom domain. Therefore, in the performed analyses, the score on the respective sleep items was subtracted from the total score to derive depression severity irrespective of sleep disturbances.

#### 4.3.2. Insomnia

Items 1–3 of the IDS-SR_30_ and the QIDS-SR_16_ assess the main night-time symptoms of insomnia pertaining to difficulty falling asleep, staying asleep, and early awakening. In line with previous research [36,55,56], the cumulative score of the three items in either scale was used as a continuous indicator of insomnia severity. This approach shows substantial concordance with assessment of insomnia severity using prospective sleep diary data [57].

#### 4.3.3. Laboratory Assessments

Serum and urine levels of a broad panel of biomarkers have been previously assessed using Enzyme-Linked Immuno-Sorbent Assays (ELISA) kits. The selection of biomarker assessments was determined in two previous studies that aimed to identify general [66] and gender-specific [75] associations of biomarker levels with MDD. The panel included 24 biomarkers pertaining to immunologic, neurotrophic, neuropeptide, neuroendocrine, and metabolic functioning. ELISA kits were acquired from various vendors, and their reported intra- and inter-assay coefficient of variation (CV) is provided in Supplementary Table S3.

For each ELISA assay, specific standard operating procedures (SOPs) were set up, which included all experimental variables to assure full experimental traceability. Each SOP was based on the instructions guide of the manufacture and contained minor tweaks with respect to extra dilution steps, the addition of calibrators, and adjustments of pipetting volumes to control for pipetting errors during dilution steps. An ELISA plate washer (Biorad PW40) was used for all washing steps. TMB absorption measurements were performed on a Microtiter plate reader (thermos Multiskan Spectrum) at 450 nm using 620 nm as a reference wavelength. Biomarker concentrations were determined by use of a 4-parameter logistic regression (4-PL) model without weight factors. To control for potential variability in the ELISA kits, all parameter samples were randomised and divided over the various ELISA plates, and each biomarker was analysed within one batch. Each ELISA plate contained Quality Control (QC) samples with known concentrations within the low and high range. These QC samples were prepared by analysing previously unused sampled followed by selecting from two to four samples within the desired range, which were pooled and aliquoted. Calibrators and QC samples were measured in duplicate, whereas samples were measured in singlicate. For both calibrators and QC samples, a CV of 15% was used as run acceptance criteria.

### 4.4. Statistical Analyses

Statistical analyses were performed using Statistical Package for the Social Sciences (SPSS) 28 [76] and R [77]. Demographic and clinical characteristics were evaluated using descriptive statistics. A linear regression analysis was performed for each biomarker to examine the association between insomnia severity (independent variable) and biomarker level (dependent variable). To evaluate the relationship between insomnia severity and biomarker levels when controlling for potential confounders, a multivariate regression analysis was performed for each biomarker with depression severity, medication use, gender, age, and cohort of origin as additional predictors of biomarker levels. The R package ‘stats’ [78] was used to adjust *p*-values in the univariate and multivariate regression models using the Benjamini–Hochberg method [79,80]. Both unadjusted and adjusted *p*-values are reported.

Dependent variables were assessed for normality prior to analyses and ln-transformation was applied to non-normal distributions. Outliers were detected according to the outlier labelling rule [81,82] and were treated as missing data. Regression diagnostics were applied to confirm that the assumptions of linear regression were not violated. Robust confidence intervals were computed for variables demonstrating heteroscedasticity in either the univariate of multivariate model [83].

Results are reported in numbers (*n*) with percentages (%), means (*M*) with standard deviation (*SD*), medians (*Mdn*) with interquartile range (IQR), unstandardized beta values (*b*) with 95% confidence intervals (95% CI), standardized beta values (β), and *R*^2^-statistic (*R*^2^). For all analyses, a two-tailed 5% significance level was adopted.

## 5. Conclusions

In conclusion, for the included biomarkers current findings do not support an influence of insomnia on the clinical pathophysiology of MDD. This is in contrast with previous findings in non-clinical populations. While more research is necessary, these divergent findings might suggest that the relationship between insomnia and pathophysiological mechanisms may pertain more to the aetiology rather than the clinical course of MDD.

## Figures and Tables

**Table 1:** Demographic and clinical characteristics.

Total Sample, *N*		227 ^a^	
Sex		*n*	%
	Female	134	59.0
	Male	92	40.5
Age		*M*	*SD*
	Years	42.04	12.63
Antidepressant use		*n*	%
	Yes	43	18.9
	No	156	68.7
Cohort of origin		*n*	%
	PIDON	38	16.7
	tPEMF	56	24.7
	MOTAR	133	58.6
Depression severity		*M*	*SD*
	QIDS-SR_16_	17.32	4.91
	QIDS-SR_16_ (sleep items excluded)	14.86	4.57
	QIDS-SR_16_ insomnia severity	4.69	2.47
Current episode duration		*n*	*%*
	Less than 1 month	96	42.3
	1–6 months	20	8.8
	6 months–1 year	11	4.8
	More than 1 year	49	21.6
Recurrence		*Mdn*	IQR
	Lifetime number of episodes	2	1–5

Note: IQR = interquartile range; tPEMF = transcranial pulsed electromagnetic fields; PIDON = Pieken in de Delta Oost Nederland; MOTAR = Mood Treatment with Antidepressants or Running; QIDS-SR_16_ = self-rated Quick Inventory of Depressive Symptomatology. ^a^ Total number of participants may differ among performed analyses due to missing data.

**Table 2:** Association between insomnia severity and levels of serum biomarkers.

	Biomarker	Unit of Measurement	*n*		*b*			*p*		
				*b*	95% CI		β			*R* ^2^
					LL	UL		Unadjusted	Adjusted	
Immunologic										
	α1-antitrypsin	mg/L	213	−0.073	−4.797	4.650	−0.002	0.976	0.988	0.000
	Calprotectin ^a^	μg/mL	216	−0.005	−0.041	0.031	−0.019	0.779	0.980	0.000
	cAMP	pmol/mL	214	−0.292	−1.058	0.475	−0.051	0.454	0.905	0.003
	Endothelin-1 ^a^	pg/mL	215	−0.006	−0.019	0.008	−0.057	0.408	0.868	0.003
	Myeloperoxidase ^a^	ng/mL	216	−0.007	−0.040	0.026	−0.028	0.687	0.937	0.001
	Resistin ^a^	ng/mL	216	−0.030	−0.051	−0.009	−0.186	0.006	0.151	0.035
	Thromboxane ^a^	ng/mL	216	−0.043	−0.102	0.016	−0.098	0.153	0.781	0.010
	TNFαR2 ^a^	ng/mL	215	0.000	−0.015	0.014	−0.001	0.988	0.988	0.000
	Zonulin ^b^	ng/mL	214	0.168	−0.071	0.407	0.100	0.168	0.781	0.010
Neurotrophic										
	BDNF	ng/mL	215	−0.112	−0.540	0.316	−0.035	0.607	0.937	0.001
	BDNF free	ng/mL	215	−0.132	−0.524	0.259	−0.046	0.506	0.905	0.002
	BDNF total	ng/mL	215	−0.012	−0.392	0.368	−0.004	0.951	0.988	0.000
	EGF	pg/mL	215	−2.205	−15.620	11.210	−0.022	0.746	0.976	0.000
Neuropeptide										
	Substance P ^a^	pg/mL	213	−0.005	−0.021	0.011	−0.041	0.549	0.934	0.002
Neuroendocrine										
	Cortisol ^b^	μg/dL	214	−0.207	−0.583	0.168	−0.075	0.278	0.781	0.006
Metabolic										
	Acetyl-L-carnitine ^a^	ng/mL	215	0.039	0.010	0.068	0.178	0.009	0.151	0.032
	Apolipoprotein A1	mg/mL	215	−0.006	−0.024	0.011	−0.048	0.485	0.905	0.002
	Leptin ^a^	ng/mL	216	0.034	−0.024	0.093	0.079	0.249	0.781	0.006
	Prolactin ^a^	μIU/mL	214	0.023	−0.014	0.060	0.084	0.222	0.781	0.007

Note: BDNF = brain-derived neurotrophic factor; cAMP = cyclic adenosine monophosphate; CI = confidence interval; EGF = epidermal growth factor; TNFαR2 = tumour necrosis factor α receptor 2. A significant *b*-coefficient indicates the β-coefficient is also significant. *b* represents unstandardised regression coefficients; β indicates standardised regression coefficients. LL and UL indicate the lower and upper limit of the confidence interval, respectively. ^a^ Ln-transformation was applied. ^b^ Heteroscedasticity-adjusted confidence interval was computed.

**Table 3:** Association between insomnia severity and levels of urine biomarkers.

	Biomarker	Unit of Measurement	*n*		*b*			*p*		
				*b*	95% CI		β			*R* ^2^
					LL	UL		Unadjusted	Adjusted	
Immunologic										
	α1-antitrypsin	μg/L	210	0.031	−0.023	0.086	0.078	0.260	0.781	0.006
	Calprotectin ^ab^	ng/mL	210	−0.005	−0.126	0.115	−0.006	0.931	0.988	0.000
	cGMP ^a^	pmol/mL	210	−0.013	−0.043	0.017	−0.059	0.391	0.868	0.004
	HVEM ^a^	ng/mL	210	−0.017	−0.050	0.015	−0.073	0.289	0.781	0.005
	Isoprostane−2 ^a^	ng/mL	209	−0.007	−0.041	0.027	−0.030	0.670	0.937	0.001
	Lipocalin−2 ^a^	ng/mL	209	0.047	−0.028	0.121	0.086	0.216	0.781	0.007
	LTB4 ^a^	pg/mL	210	−0.007	−0.036	0.021	−0.035	0.612	0.937	0.001
	Resistin ^a^	ng/mL	209	−0.035	−0.087	0.016	−0.094	0.176	0.781	0.009
	Thromboxane ^a^	ng/mL	210	−0.001	−0.036	0.034	−0.005	0.947	0.988	0.000
Neurotrophic										
	EGF ^a^	ng/mL	209	−0.030	−0.064	0.003	−0.123	0.077	0.781	0.015
	Midkine ^ab^	pg/mL	207	0.005	−0.040	0.049	0.017	0.832	0.988	0.000
Neuropeptide										
	Substance P ^b^	pg/mL	205	−2.503	−8.435	3.428	−0.058	0.406	0.868	0.003
Neuroendocrine										
	Aldosterone ^a^	ng/mL	207	−0.019	−0.054	0.017	−0.073	0.299	0.781	0.077
	Cortisol ^ab^	μg/dL	209	−0.010	−0.059	0.039	−0.032	0.689	0.937	0.001
Metabolic										
	Acetyl-L-carnitine ^a^	ng/mL	210	−0.003	−0.043	0.037	−0.010	0.883	0.988	0.000

Note: cGMP = cyclic guanosine monophosphate; CI = confidence interval; EGF = epidermal growth factor; HVEM = herpes virus entry mediator; LTB4 = leukotriene B4. A significant *b*-coefficient indicates the β-coefficient is also significant. *b* represents unstandardised regression coefficients; β indicates standardised regression coefficients. LL and UL indicate the lower and upper limit of the confidence interval, respectively. ^a^ Ln-transformation was applied. ^b^ Heteroscedasticity-adjusted confidence interval was computed.

**Table 4:** Association between insomnia severity and levels of serum biomarkers adjusted for relevant covariates ^a^.

	Biomarker	Unit of Measurement	*n*		*b*			*p*		
				*b*	95% CI		β			*R* ^2^
					LL	UL		Unadjusted	Adjusted	
Immunologic										
	α1-antitrypsin	mg/L	175	−0.717	−7.121	5.686	−0.020	0.825	0.935	0.040
	Calprotectin ^b^	μg/mL	178	0.021	−0.020	0.062	0.091	0.311	0.906	0.025
	cAMP	pmol/mL	176	−0.290	−1.223	0.644	−0.050	0.541	0.906	0.196
	Endothelin-1 ^b^	pg/mL	177	−0.019	−0.037	−0.002	−0.183	0.033	0.528	0.119
	Myeloperoxidase ^b^	ng/mL	178	0.010	−0.034	0.055	0.041	0.650	0.906	0.021
	Resistin ^b^	ng/mL	178	−0.016	−0.045	0.013	−0.096	0.266	0.906	0.104
	Thromboxane ^b^	ng/mL	178	−0.030	−0.109	0.049	−0.067	0.448	0.906	0.044
	TNFαR2 ^b^	ng/mL	177	−0.018	−0.038	0.001	−0.166	0.062	0.528	0.050
	Zonulin ^c^	ng/mL	176	−0.123	−0.433	0.187	−0.070	0.435	0.906	0.176
Neurotrophic										
	BDNF	ng/mL	178	−0.148	−0.725	0.430	−0.044	0.615	0.906	0.054
	BDNF free	ng/mL	178	−0.225	−0.736	0.286	−0.077	0.386	0.906	0.049
	BDNF total	ng/mL	178	−0.106	−0.617	0.406	−0.036	0.684	0.906	0.043
	EGF	pg/mL	177	−5.792	−24.025	12.441	−0.056	0.531	0.906	0.063
Neuropeptide										
	Substance P ^b^	pg/mL	175	−0.009	−0.031	0.013	−0.071	0.434	0.906	0.013
Neuroendocrine										
	Cortisol ^c^	μg/dL	176	0.056	−0.404	0.517	0.019	0.809	0.935	0.192
Metabolic										
	Acetyl-L-carnitine ^b^	ng/mL	178	0.036	0.000	0.073	0.167	0.049	0.528	0.134
	Apolipoprotein A1	mg/mL	177	0.001	−0.021	0.023	0.006	0.942	0.965	0.060
	Leptin ^b^	ng/mL	178	−0.020	−0.083	0.044	−0.043	0.539	0.906	0.399
	Prolactin ^b^	μIU/mL	178	0.009	−0.030	0.048	0.036	0.653	0.906	0.205

Note: BDNF = brain-derived neurotrophic factor; cAMP = cyclic adenosine monophosphate; EGF = epidermal growth factor; CI = confidence interval; TNFαR2 = tumour necrosis factor α receptor 2. A significant *b*-coefficient indicates the β-coefficient is also significant. *b* represents unstandardised regression coefficients; β indicates standardised regression coefficients. LL and UL indicate the lower and upper limit of the confidence interval, respectively. ^a^ Covariates included in the model: non-sleep depression severity, antidepressant use, gender, age, and cohort of origin. ^b^ Ln-transformation was applied. ^c^ Heteroscedasticity-adjusted confidence interval was computed.

**Table 5:** Association between insomnia severity and levels of urine biomarkers adjusted for relevant covariates ^a^.

	Biomarker	Unit of Measurement	*n*		*b*			*p*		
				*b*	95% CI		β			*R* ^2^
					LL	UL		Unadjusted	Adjusted	
Immunologic										
	α1-antitrypsin	μg/L	174	0.025	−0.041	0.091	0.060	0.454	0.906	0.256
	Calprotectin ^bc^	ng/mL	174	−0.017	−0.119	0.084	−0.020	0.735	0.906	0.564
	cGMP ^b^	pmol/mL	174	−0.007	−0.045	0.031	−0.030	0.729	0.906	0.095
	HVEM ^b^	ng/mL	174	−0.010	−0.050	0.031	−0.040	0.645	0.906	0.118
	Isoprostane−2 ^b^	ng/mL	173	0.008	−0.035	0.051	0.032	0.713	0.906	0.113
	Lipocalin−2 ^b^	ng/mL	173	0.073	−0.018	0.164	0.127	0.115	0.784	0.254
	LTB4 ^b^	pg/mL	174	0.001	−0.035	0.037	0.005	0.959	0.965	0.063
	Resistin ^b^	ng/mL	173	−0.012	−0.079	0.055	−0.031	0.719	0.906	0.109
	Thromboxane ^b^	ng/mL	174	0.003	−0.041	0.047	0.011	0.901	0.965	0.145
Neurotrophic										
	EGF ^b^	ng/mL	174	−0.007	−0.048	0.034	−0.027	0.746	0.906	0.183
	Midkine ^bc^	pg/mL	172	0.025	−0.026	0.076	0.084	0.336	0.906	0.221
Neuropeptide										
	Substance P ^c^	pg/mL	170	−1.710	−8.455	5.034	−0.038	0.617	0.906	0.294
Neuroendocrine										
	Aldosterone ^b^	ng/mL	171	0.001	−0.044	0.046	0.004	0.965	0.965	0.074
	Cortisol ^bc^	μg/dL	174	0.020	−0.038	0.078	0.064	0.508	0.906	0.057
Metabolic										
	Acetyl-L-carnitine ^b^	ng/mL	174	−0.048	−0.096	0.001	−0.168	0.055	0.528	0.105

Note: cGMP = cyclic guanosine monophosphate; CI = confidence interval; EGF = epidermal growth factor; HVEM = herpes virus entry mediator; LTB4 = leukotriene B4. A significant *b*-coefficient indicates the β-coefficient is also significant. *b* represents unstandardised regression coefficients; β indicates standardised regression coefficients. LL and UL indicate the lower and upper limit of the confidence interval, respectively. ^a^ Covariates included in the model: non-sleep depression severity, antidepressant use, gender, age, and cohort of origin. ^b^ Ln-transformation was applied. ^c^ Heteroscedasticity-adjusted confidence interval was computed.

## Data Availability

The data presented in this study are available on request from the corresponding author. The data are not publicly available due to privacy.

## Supplementary Materials

The following supporting information can be downloaded at: https://www.mdpi.com/article/10.3390/ijms24098437/s1.
