# Supplementary material for: The Relationship between Insomnia and the Pathophysiology of Major Depressive Disorder: An Evaluation of a Broad Selection of Serum and Urine Biomarkers
Source: Int J Mol Sci. 2023 May 8;24(9):8437. doi: 10.3390/ijms24098437 (PMC10179282; doi:10.3390/ijms24098437)
Supplement: Supplementary file 1 [file ijms-24-08437-s001.zip › ijms-2283189-supplementary.pdf]

Table S1. Associations of insomnia severity and relevant covariates with levels of serum biomarkers.

Biomarker level	Unit of measurement	*n*	Effect	*b*	*b* 95% CI LL	*b* 95% CI UL	β	*p*	VIF
Immunologic									
α1-antitrypsin	mg/L	175							
			Insomnia severity	-.717	-7.121	5.686	-.020	.825	1.402
			Depression severity	.755	-2.659	4.170	.040	.663	1.433
			Antidepressant use	-3.457	-51.358	44.444	-.016	.887	2.125
			Gender	18.049	-8.856	44.954	.103	.187	1.061
			Age	-.964	-2.195	.267	-.136	.124	1.347
			Cohort of origin	-15.318	-51.873	21.237	-.091	.409	2.128
Calprotectin [a]	μg/mL	178							
			Insomnia severity	.021	-.020	.062	.091	.311	1.402
			Depression severity	-.002	-.025	.020	-.020	.825	1.441
			Antidepressant use	.232	-.075	.539	.165	.138	2.142
			Gender	.014	-.159	.187	.013	.871	1.064
			Age	.001	-.007	.009	.031	.726	1.349
			Cohort of origin	.115	-.121	.351	.106	.338	2.154
cAMP	pmol/mL	176							
			Insomnia severity	-.290	-1.223	.644	-.050	.541	1.400
			Depression severity	-.061	-.569	.447	-.020	.812	1.427
			Antidepressant use	1.464	-5.547	8.475	.042	.681	2.139
			Gender	-11.242	-15.227	-7.257	-.397	.000	1.067
			Age	.119	-.065	.303	.102	.204	1.340
			Cohort of origin	.189	-5.208	5.585	.007	.945	2.149
Endothelin-1 [a]	pg/mL	177							
			Insomnia severity	-.019	-.037	-.002	-.183	.033	1.404
			Depression severity	.009	-.001	.019	.161	.065	1.446
			Antidepressant use	-.013	-.146	.120	-.021	.846	2.140
			Gender	-.065	-.140	.010	-.126	.091	1.063
			Age	.007	.003	.010	.324	.000	1.348
			Cohort of origin	.104	.002	.207	.212	.046	2.149
Myeloperoxidase [a]	ng/mL	178							
			Insomnia severity	.010	-.034	.055	.041	.650	1.402
			Depression severity	.000	-.024	.024	.000	.996	1.441
			Antidepressant use	.286	-.051	.622	.186	.095	2.142
			Gender	-.006	-.195	.184	-.005	.953	1.064
			Age	-.004	-.012	.005	-.074	.400	1.349
			Cohort of origin	.089	-.170	.348	.075	.499	2.154
Resistin [a]	ng/mL	178							
			Insomnia severity	-.016	-.045	.013	-.096	.266	1.402
			Depression severity	-.007	-.023	.009	-.077	.377	1.441
			Antidepressant use	.042	-.175	.259	.040	.703	2.142
			Gender	.073	-.049	.196	.088	.238	1.064

				Age	-.009	-.015	-.004	-.273	.001	1.349
				Cohort of origin	-.009	-.176	.158	-.012	.912	2.154
	Thromboxane [a]	ng/mL	178							
				Insomnia severity	-.030	-.109	.049	-.067	.448	1.402
				Depression severity	.001	-.042	.043	.002	.978	1.441
				Antidepressant use	.485	-.108	1.078	.176	.109	2.142
				Gender	-.254	-.588	.080	-.116	.136	1.064
				Age	-.012	-.027	.003	-.134	.124	1.349
				Cohort of origin	.349	-.107	.805	.166	.133	2.154
	TNFαR2 [a]	ng/mL	177							
				Insomnia severity	-.018	-.038	.001	-.166	.062	1.406
				Depression severity	.008	-.002	.019	.141	.116	1.437
				Antidepressant use	.027	-.119	.172	.039	.719	2.140
				Gender	-.074	-.156	.008	-.136	.078	1.060
				Age	.002	-.002	.006	.079	.362	1.346
				Cohort of origin	-.004	-.116	.108	-.008	.939	2.150
	Zonulin [b]	ng/mL	176							
				Insomnia severity	-.123	-.433	.187	-.070	.435	1.418
				Depression severity	.223	.065	.380	.238	.006	1.449
				Antidepressant use	.214	-2.096	2.524	.020	.855	2.141
				Gender	-.854	-2.022	.314	-.099	.151	1.066
				Age	.118	.062	.173	.333	.000	1.384
				Cohort of origin	-.555	-2.378	1.268	-.068	.548	2.161
Neurotrophic										
	BDNF	ng/mL	178							
				Insomnia severity	-.148	-.725	.430	-.044	.615	1.402
				Depression severity	.029	-.283	.340	.016	.856	1.441
				Antidepressant use	.828	-3.509	5.166	.041	.707	2.142
				Gender	-.161	-2.606	2.285	-.010	.897	1.064
				Age	.086	-.027	.198	.130	.134	1.349
				Cohort of origin	-1.787	-5.123	1.549	-.115	.292	2.154
	BDNF free	ng/mL	178							
				Insomnia severity	-.225	-.736	.286	-.077	.386	1.402
				Depression severity	.045	-.230	.321	.029	.745	1.441
				Antidepressant use	-.429	-4.266	3.408	-.024	.826	2.142
				Gender	.343	-1.821	2.506	.024	.755	1.064
				Age	.081	-.018	.180	.140	.109	1.349
				Cohort of origin	-1.976	-4.927	.976	-.145	.188	2.154
	BDNF total	ng/mL	178							
				Insomnia severity	-.106	-.617	.406	-.036	.684	1.402
				Depression severity	.068	-.208	.343	.043	.629	1.441
				Antidepressant use	.380	-3.458	4.218	.021	.845	2.142
				Gender	.038	-2.126	2.202	.003	.972	1.064
				Age	.076	-.024	.175	.131	.134	1.349
				Cohort of origin	-1.420	-4.372	1.533	-.104	.344	2.154
	EGF	pg/mL	177							

			Insomnia severity	-5.792	-24.025	12.441	-.056	.531	1.424
			Depression severity	4.173	-5.637	13.984	.076	.402	1.468
			Antidepressant use	77.142	-58.631	212.914	.123	.264	2.180
			Gender	-36.102	-111.968	39.764	-.072	.349	1.060
			Age	-2.582	-6.063	.899	-.126	.145	1.340
			Cohort of origin	119.375	14.808	223.943	.247	.026	2.179
Neuropeptide									
Substance P [a]	pg/mL	175							
			Insomnia severity	-.009	-.031	.013	-.071	.434	1.405
			Depression severity	.006	-.006	.018	.084	.361	1.435
			Antidepressant use	-.048	-.213	.117	-.064	.566	2.134
			Gender	.019	-.075	.114	.032	.686	1.082
			Age	.000	-.005	.004	-.020	.827	1.355
			Cohort of origin	-.014	-.141	.113	-.025	.826	2.142
Neuroendocrine									
Cortisol [b]	μg/dL	176							
			Insomnia severity	.056	-.404	.517	.019	.809	1.405
			Depression severity	-.108	-.357	.141	-.069	.392	1.431
			Antidepressant use	-2.017	-5.359	1.324	-.114	.235	2.139
			Gender	.144	-1.804	2.093	.010	.884	1.061
			Age	-.107	-.202	-.013	-.183	.026	1.349
			Cohort of origin	3.247	.564	5.930	.238	.018	2.147
Metabolic									
Acetyl-L-carnitine [a]	ng/mL	178							
			Insomnia severity	.036	.000	.073	.167	.049	1.402
			Depression severity	-.013	-.033	.006	-.115	.181	1.441
			Antidepressant use	.277	.004	.549	.209	.047	2.142
			Gender	.055	-.099	.208	.052	.484	1.064
			Age	.002	-.005	.009	.038	.648	1.349
			Cohort of origin	-.149	-.358	.061	-.146	.163	2.154
Apolipoprotein A1	mg/mL	177							
			Insomnia severity	.001	-.021	.023	.006	.942	1.399
			Depression severity	-.002	-.014	.010	-.027	.764	1.441
			Antidepressant use	-.071	-.235	.094	-.092	.398	2.140
			Gender	.145	.052	.238	.236	.002	1.064
			Age	.002	-.003	.006	.060	.486	1.347
			Cohort of origin	-.071	-.197	.056	-.120	.272	2.150
Leptin [a]	ng/mL	178							
			Insomnia severity	-.020	-.083	.044	-.043	.539	1.402
			Depression severity	.035	.001	.069	.145	.043	1.441
			Antidepressant use	.525	.049	1.001	.189	.031	2.142
			Gender	1.333	1.065	1.601	.600	.000	1.064
			Age	.016	.004	.029	.179	.010	1.349
			Cohort of origin	.298	-.068	.664	.140	.110	2.154
Prolactin [a]	μIU/mL	178							
			Insomnia severity	.009	-.030	.048	.036	.653	1.402

Depression severity	.001	-.020	.022	.007	.930	1.441
Antidepressant use	.311	.019	.604	.210	.037	2.142
Gender	.199	.034	.364	.167	.019	1.064
Age	-.014	-.022	-.006	-.289	.000	1.349
Cohort of origin	.372	.147	.597	.327	.001	2.154

Note. BDNF = brain-derived neurotrophic factor; cAMP = cyclic adenosine monophosphate; EGF = epidermal growth factor; LL = lower limit; TNFαR2 = tumour necrosis factor α receptor 2; UL = upper limit; VIF = variance inflation factor. A significant *b*-coefficient indicates the β-coefficient is also significant. *b* represents unstandardised regression coefficients; β indicates standardised regression coefficients.

[a] Ln-transformation was applied.

[b] Heteroscedasticity-adjusted confidence interval was computed.

Table S2. Associations of insomnia severity and relevant covariates with levels of urine biomarkers.

Biomarker level	Unit of measurement	*n*	Effect	*b*	*b* 95% CI LL	*b* 95% CI UL	β	*p*	VIF
Immunologic									
α1-antitrypsin [a]	μg/L	174							
			Insomnia severity	.025	-.041	.091	.060	.454	1.415
			Depression severity	.018	-.016	.051	.082	.302	1.404
			Antidepressant use	.106	-.375	.587	.044	.664	2.340
			Gender	.273	.002	.545	.136	.048	1.045
			Age	.003	-.009	.015	.040	.590	1.258
			Cohort of origin	-.721	-1.048	-.393	-.436	.000	2.256
Calprotectin [a b]	ng/mL	174							
			Insomnia severity	-.017	-.119	.084	-.020	.735	1.415
			Depression severity	.045	-.007	.096	.099	.089	1.404
			Antidepressant use	.017	-.727	.761	.003	.963	2.340
			Gender	2.825	2.379	3.271	.672	.000	1.045
			Age	.008	-.014	.030	.049	.454	1.258
			Cohort of origin	-1.148	-1.607	-.690	-.333	.000	2.256
cGMP [a]	pmol/mL	174							
			Insomnia severity	-.007	-.045	.031	-.030	.729	1.415
			Depression severity	.011	-.009	.030	.093	.286	1.404
			Antidepressant use	-.290	-.569	-.011	-.231	.042	2.340
			Gender	-.075	-.232	.083	-.071	.350	1.045
			Age	-.007	-.014	.000	-.154	.064	1.258
			Cohort of origin	-.314	-.505	-.124	-.361	.001	2.256
HVEM [a]	ng/mL	174							
			Insomnia severity	-.010	-.050	.031	-.040	.645	1.415
			Depression severity	.011	-.010	.032	.088	.306	1.404
			Antidepressant use	-.110	-.408	.188	-.081	.468	2.340
			Gender	-.254	-.422	-.086	-.221	.003	1.045
			Age	-.007	-.014	.001	-.139	.089	1.258
			Cohort of origin	-.278	-.481	-.075	-.295	.008	2.256
Isoprostane-2 [a]	ng/mL	173							
			Insomnia severity	.008	-.035	.051	.032	.713	1.420
			Depression severity	.007	-.015	.029	.053	.545	1.408
			Antidepressant use	.025	-.291	.341	.018	.874	2.339
			Gender	-.338	-.517	-.159	-.279	.000	1.042
			Age	-.009	-.018	-.001	-.190	.022	1.266
			Cohort of origin	-.137	-.353	.078	-.138	.209	2.254
Lipocalin-2 [a]	ng/mL	173							
			Insomnia severity	.073	-.018	.164	.127	.115	1.426
			Depression severity	.021	-.025	.068	.072	.370	1.417
			Antidepressant use	-.074	-.738	.590	-.022	.827	2.337
			Gender	.743	.367	1.120	.267	.000	1.044

				Age	.004	-.013	.021	.038	.611	1.268
				Cohort of origin	-.968	-1.420	-.515	-.425	.000	2.251
	LTB4 [a]	pg/mL	174							
				Insomnia severity	.001	-.035	.037	.005	.959	1.415
				Depression severity	.008	-.010	.027	.078	.383	1.404
				Antidepressant use	-.255	-.522	.011	-.217	.060	2.340
				Gender	-.195	-.346	-.045	-.196	.011	1.045
				Age	-.001	-.008	.006	-.020	.808	1.258
				Cohort of origin	-.157	-.338	.025	-.192	.090	2.256
	Resistin [a]	ng/mL	173							
				Insomnia severity	-.012	-.079	.055	-.031	.719	1.413
				Depression severity	.009	-.025	.043	.046	.596	1.397
				Antidepressant use	-.148	-.636	.340	-.067	.550	2.337
				Gender	-.295	-.571	-.018	-.158	.037	1.048
				Age	-.017	-.029	-.005	-.222	.008	1.263
				Cohort of origin	-.442	-.775	-.110	-.289	.009	2.253
	Thromboxane [a]	ng/mL	174							
				Insomnia severity	.003	-.041	.047	.011	.901	1.415
				Depression severity	.009	-.014	.032	.066	.438	1.404
				Antidepressant use	-.022	-.346	.301	-.015	.892	2.340
				Gender	-.349	-.532	-.167	-.276	.000	1.045
				Age	-.006	-.015	.002	-.125	.122	1.258
				Cohort of origin	-.283	-.504	-.063	-.273	.012	2.256
Neurotrophic										
	EGF [a]	ng/mL	174							
				Insomnia severity	-.007	-.048	.034	-.027	.746	1.415
				Depression severity	.009	-.012	.030	.072	.385	1.404
				Antidepressant use	-.023	-.322	.277	-.016	.881	2.340
				Gender	-.088	-.257	.081	-.074	.305	1.045
				Age	-.019	-.027	-.012	-.398	.000	1.258
				Cohort of origin	-.237	-.441	-.033	-.242	.023	2.256
	Midkine [a b]	pg/mL	172							
				Insomnia severity	.025	-.026	.076	.084	.336	1.413
				Depression severity	.004	-.021	.029	.025	.760	1.408
				Antidepressant use	-.147	-.500	.207	-.088	.414	2.338
				Gender	-.634	-.842	-.426	-.447	.000	1.046
				Age	-.011	-.020	-.001	-.184	.030	1.271
				Cohort of origin	-.067	-.299	.165	-.058	.569	2.253
Neuropeptide										
	Substance P [b]	pg/mL	170							
				Insomnia severity	-1.710	-8.455	5.034	-.038	.617	1.416
				Depression severity	2.896	-.736	6.527	.125	.117	1.387
				Antidepressant use	-43.715	-112.676	25.247	-.168	.212	2.376
				Gender	-32.718	-62.808	-2.628	-.150	.033	1.045
				Age	-.322	-1.589	.946	-.037	.617	1.263
				Cohort of origin	-110.077	-160.200	-59.954	-.618	.000	2.303

Neuroendocrine									
Aldosterone [a]	ng/mL	171							
			Insomnia severity	.001	-.044	.046	.004	.965	1.403
			Depression severity	.003	-.020	.027	.026	.773	1.406
			Antidepressant use	.142	-.195	.478	.096	.408	2.365
			Gender	.093	-.096	.281	.074	.333	1.044
			Age	-.013	-.021	-.005	-.257	.003	1.256
			Cohort of origin	.004	-.224	.232	.004	.970	2.278
Cortisol [a b]	μg/dL	174							
			Insomnia severity	.020	-.038	.078	.064	.508	1.415
			Depression severity	-.014	-.040	.013	-.089	.301	1.404
			Antidepressant use	-.151	-.610	.308	-.088	.517	2.340
			Gender	-.095	-.323	.134	-.065	.416	1.045
			Age	-.013	-.023	-.002	-.211	.015	1.258
			Cohort of origin	-.036	-.319	.247	-.031	.799	2.256
Metabolic									
Acetyl-L-carnitine [a]	ng/mL	174							
			Insomnia severity	-.048	-.096	.001	-.168	.055	1.415
			Depression severity	.032	.007	.057	.219	.012	1.404
			Antidepressant use	.205	-.150	.561	.128	.256	2.340
			Gender	-.153	-.354	.048	-.113	.134	1.045
			Age	-.001	-.010	.008	-.009	.909	1.258
			Cohort of origin	-.117	-.359	.125	-.105	.342	2.256

Note. cGMP = cyclic guanosine monophosphate; EGF = epidermal growth factor; HVEM = herpes virus entry mediator; LL = lower limit; LTB4 = leukotriene B4; UL = upper limit; VIF = variance inflation factor. A significant *b*-coefficient indicates the β-coefficient is also significant. *b* represents unstandardised regression coefficients; β indicates standardised regression coefficients.

[a] Ln-transformation was applied.

[b] Heteroscedasticity-adjusted confidence interval was computed.

Table S3. Intra- and inter-assay reliability as reported by vendors of assay kits used for biomarker measurement.

Biomarker	Sample(s)	Vendor	Intra-assay CV	Inter-assay CV
Immunologic				
α1-antitrypsin	Serum and urine	Immundiagnostik GmbH, Bensheim, Germany	<8%	<11%
Calprotectin	Serum and urine	Sanbio B, Hycult biotech, Uden, The Netherlands	<6%	<5%
cAMP	Serum	R&D systems Europe Ltd., Abingdon, United Kingdom	<4%	<9%
cGMP	Urine	R&D systems Europe Ltd., Abingdon, United Kingdom	<7%	<9%
Endothelin-1	Serum	R&D systems Europe Ltd., Abingdon, United Kingdom	<3%	<7%
HVEM	Urine	Ray Biotech Inc., Norcross, GA, USA	<10%	<12%
Isoprostane-2	Urine	Northwest Life Science Specialties, LLC, Vancouver, WA, USA	U/A	U/A
Lipocalin-2	Urine	Ray Biotech Inc., Norcross, GA, USA	<10%	<12%
LTB4	Urine	R&D systems Europe Ltd., Abingdon, United Kingdom	<5%	<9%
Myeloperoxidase	Serum	R&D systems Europe Ltd., Abingdon, United Kingdom	<3%	<10%
Resistin	Serum and urine	R&D systems Europe Ltd., Abingdon, United Kingdom	<5%	<9%
Thromboxane	Serum and urine	R&D systems Europe Ltd., Abingdon, United Kingdom	<6%	<7%
TNFαR2	Serum	Ray Biotech Inc., Norcross, GA, USA	<10%	<12%
Zonulin	Serum	Immundiagnostik GmbH, Bensheim, Germany	<5%	<9%
Neurotrophic				
BDNF	Serum	Promega Benelux BV, Leiden, The Netherlands	U/A	U/A
BDNF free	Serum	Promega Benelux BV, Leiden, The Netherlands	U/A	U/A
BDNF total	Serum	Promega Benelux BV, Leiden, The Netherlands	U/A	U/A
EGF	Serum and urine	Ray Biotech Inc., Norcross, GA, USA	<10%	<12%
Midkine	Urine	Cellmid Limited, Perth, Australia	U/A	U/A
Neuropeptide				
Substance P	Serum and urine	R&D systems Europe Ltd., Abingdon, United Kingdom	<7%	<12%
Neuroendocrine				
Aldosterone	Urine	LDN, Germany	<6%	<8%
Cortisol	Serum and urine	R&D systems Europe Ltd., Abingdon, United Kingdom	<7%	<14%
Metabolic				
Acetyl-L-carnitine	Serum and urine	Abbexa Ltd, United Kingdom	<10%	<12%
Apolipoprotein A1	Serum	R&D systems Europe Ltd., Abingdon, United Kingdom	<4%	<7%
Leptin	Serum	Ray Biotech Inc., Norcross, GA, USA	<10%	<12%
Prolactin	Serum	Diagnostics Biochem Canada Inc.	<10%	<12%

Note. BDNF = brain-derived neurotrophic factor; cAMP = cyclic adenosine monophosphate; cGMP = cyclic guanosine monophosphate; EGF = epidermal growth factor; HVEM = herpes virus entry mediator; LTB4 = leukotriene B4; TNFαR2 = tumour necrosis factor α receptor 2; U/A = unavailable.
